# Impaired fertility and motor function in a zebrafish model for classic galactosemia

**DOI:** 10.1007/s10545-017-0071-1

**Published:** 2017-09-14

**Authors:** Jo M. Vanoevelen, Britt van Erven, Jörgen Bierau, Xiaoping Huang, Gerard T. Berry, Rein Vos, Ana I. Coelho, M. Estela Rubio-Gozalbo

**Affiliations:** 1 Department of Clinical Genetics, Maastricht University Medical Centre, Universiteitssingel 50, P.O. Box 616, box 16, 6200 MD Maastricht, The Netherlands; 2 GROW-School for Oncology and Developmental Biology, Maastricht University Medical Centre, Maastricht, The Netherlands; 3 Department of Pediatrics, Maastricht University Medical Centre, Maastricht, The Netherlands; 4 The Manton Center for Orphan Disease Research, Division of Genetics and Genomics, Boston Children’s Hospital, Harvard Medical School, Boston, MA USA; 5 Department of Methodology and Statistics, School for Public Health and Primary Care (CAPHRI), Maastricht University, Maastricht, The Netherlands

## Abstract

Classic galactosemia is a genetic disorder of galactose metabolism, caused by severe deficiency of galactose-1-phosphate uridylyltransferase (GALT) enzyme activity due to mutations of the *GALT* gene. Its pathogenesis is still not fully elucidated, and a therapy that prevents chronic impairments is lacking. In order to move research forward, there is a high need for a novel animal model, which allows organ studies throughout development and high-throughput screening of pharmacologic compounds. Here, we describe the generation of a *galt* knockout zebrafish model and present its phenotypical characterization. Using a TALEN approach, a *galt* knockout line was successfully created. Accordingly, biochemical assays confirm essentially undetectable galt enzyme activity in homozygotes. Analogous to humans, *galt* knockout fish accumulate galactose-1-phosphate upon exposure to exogenous galactose. Furthermore, without prior exposure to exogenous galactose, they exhibit reduced motor activity and impaired fertility (lower egg quantity per mating, higher number of unsuccessful crossings), resembling the human phenotype(s) of neurological sequelae and subfertility. In conclusion, our *galt* knockout zebrafish model for classic galactosemia mimics the human phenotype(s) at biochemical and clinical levels. Future studies in our model will contribute to improved understanding and management of this disorder.

**Electronic supplementary material:**

The online version of this article (doi:10.1007/s10545-017-0071-1) contains supplementary material, which is available to authorized users.

## Introduction

Classic galactosemia is a genetic disorder of galactose metabolism, in which there is a severe impairment of galactose-1-phosphate uridylyltransferase activity (GALT). Patients present during the neonatal period with a toxic syndrome, after ingestion of galactose-containing milk. Though a galactose-restricted diet is life-saving during this phase, it fails to prevent the development of chronic impairments affecting mainly brain and gonads (Antshel et al 2004; Doyle et al 2010; Gubbels et al 2011; Kaufman et al 1986; Potter et al 2013; Rubio-Agusti et al 2013; Schadewaldt et al 2010; Timmers et al 2012, 2015; Waisbren et al 1983). To date, the exact pathogenic pathways and their onset are still not fully elucidated. Accumulation of toxic galactose metabolites, such as galactose-1-phosphate (Gal-1-P) and galactitol (Ross et al 2004; Schadewaldt et al 2003; Slepak et al 2005; Yager et al 2003), aberrant glycosylation (Coss et al 2014; Maratha et al 2016), endoplasmic reticulum (ER) stress (Slepak et al 2007; Tang et al 2014), GALT misfolding (Coelho et al 2014; McCorvie et al 2013), and dysregulation of phosphoinositide 3 kinase/protein kinase B (PI3K/Akt) signaling pathway (Balakrishnan et al 2016) have been implicated.

Animal models for classic galactosemia that tried to mimic the biochemical and clinical human phenotype(s) have been developed, a fruit fly (Kushner et al 2010) and two mouse models (Leslie et al 1996; Tang et al 2014), which have shown their value (Balakrishnan et al 2016; Chen et al 2017; Daenzer et al 2016; Jumbo-Lucioni et al 2013, 2014, b, 2016). We have created a zebrafish model for classic galactosemia that complements these models. The zebrafish model is an ideal intermediate between the invertebrate and the mammalian model system since it is, as a vertebrate, evolutionary more closely related to humans while being a more high-throughput system (high amounts of offspring). Additionally, embryos can be used as a replacement for adult fish, and embryonic development is external, facilitating studies in the prenatal stages. Due to their transparency and amiability to genetic modification, different organ systems are easy to study in zebrafish using reporter lines.

Here, we describe the development of a *galt* knockout zebrafish model and present its phenotypical characterization. This model is meant to improve knowledge on the developmental stage at which lesions occur and for the development of new therapeutic strategies.

## Materials and methods

### Ethics statement

This study was approved by the Animal Ethics Committee of the University of Maastricht (*Dier Experimenten Commissie*, Maastricht University) and the Dutch National Central Authority for Scientific Procedures on Animals (*Centrale Commissie Dierproeven*) (AVD107002016545). In addition, approval was obtained from the Animal Ethics Committee of the University of Liège (*Commission d’Ethique de l’Utilization des Animaux*, Université de Liège, Liège, Belgium; dossier 1576) for the experiments we performed initially in the GIGA Zebrafish Facility, Liège. At all times, the care and use of animals were in agreement with the national and local guidelines. Care of animals and animal experiments were conducted exclusively by licensed staff.

### Husbandry

Zebrafish (*Danio rerio*) were housed in recirculating systems on a 14/10 day-night regime. Husbandry was essentially performed as described by Lawrence (2007).

### Generation of galt-deficient zebrafish

The design and assembly of the TALE nuclease (TALEN) construct targeting the *galt* gene (ENSDARG00000069543) to create a knockout was based on the protocol described by Cermak et al (2011). TALEN coding sequences were obtained from TAL Effector-Nucleotide Targeter (Bogdanove Laboratory, Cornell University; (Cermak et al 2011; Doyle et al 2012)). In order to ensure loss-of-function of the galt protein, TALEN sequences located within the first part of the gene were chosen. The DNA binding sites for the TALEN pair targeting *galt* were: left site 5′-TTTTGGTCTCGGCCCATCGG-3′, right site 5′-AAAGGACAGGTGGAGAAA-3′. Constructs were assembled using the Golden Gate TALEN and Tal Effector Kit 2.0 (Addgene) (Cermak et al 2011). The integrity of the final constructs was verified by Sanger sequencing. The final TALEN plasmids were linearized and used as a template for in vitro mRNA synthesis using the Ambion mMESSAGE mMACHINE T3 Transcription Kit (Life Technologies).

**Fig. 1:**
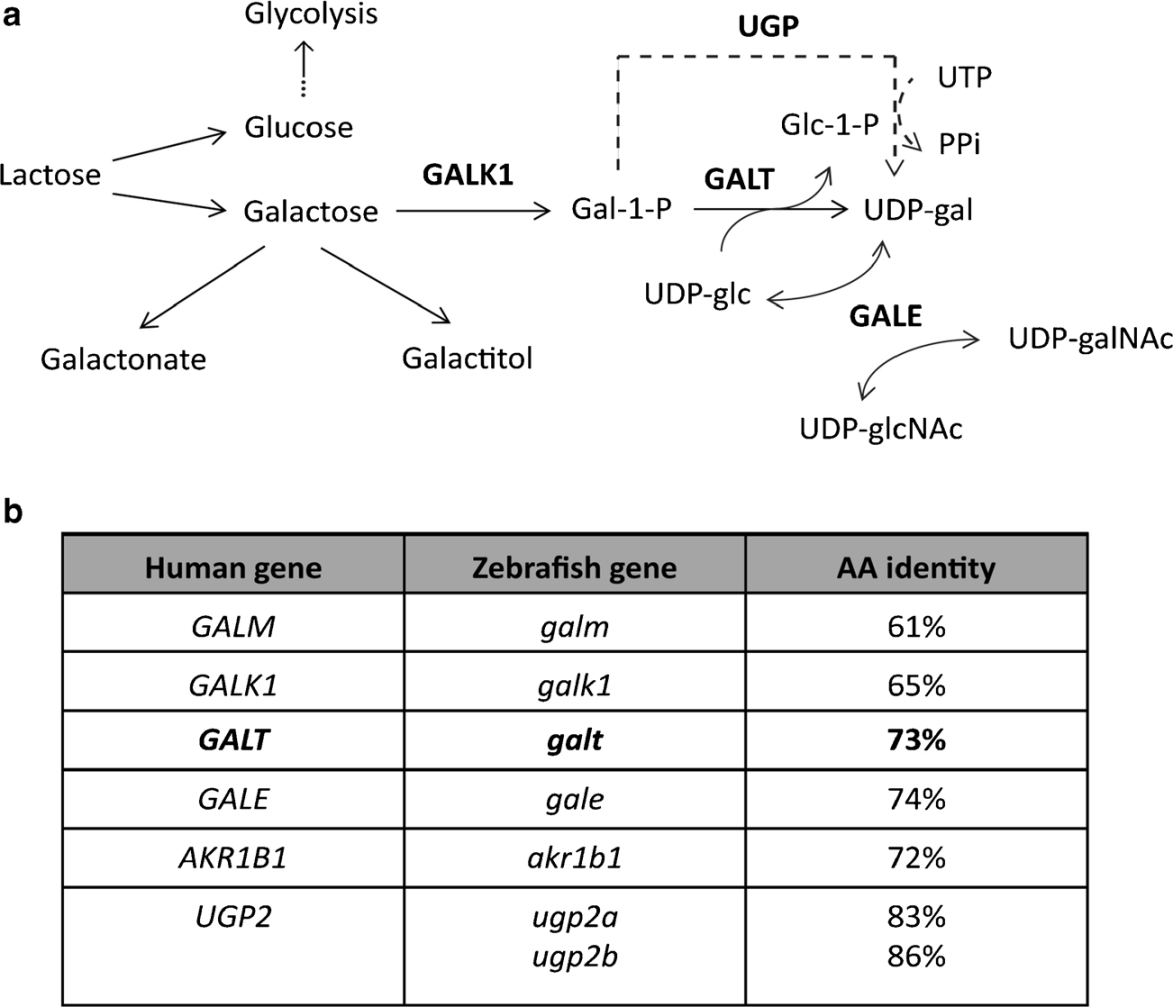
Galactose metabolism in humans and zebrafish. Genes encoding the enzymes involved in galactose metabolism (panel **a**) in humans show strong conservation with their zebrafish counterparts (panel **b**). Strikingly, only the *UGP2* gene is duplicated in zebrafish (*ugp2a* and *ugp2b*). GALK1: galactokinase, GALT: Galactose-1-phosphate uridylyltransferase, GALE: UDP-galactose 4′-epimerase, UGP: UDP-glucose/UDP-galactose pyrophosphorylase, Panel **b** shows the percentage of amino acid similarity between the human and zebrafish homologs of the enzymes involved in galactose metabolism

Subsequently, the mRNA of both left and right TALEN constructs were injected in wildtype zebrafish embryos in one-cell stage. Injected fish were raised to adulthood and outcrossed, followed by confirmation of heterozygotes in the offspring. Heterozygous fish were incrossed in order to generate homozygous, *galt* knockout zebrafish (Fig. 1; Supplementary Figs. S1 and S2).

### Genotyping

Zebrafish embryos were euthanized with a lethal dose of tricaine. Biopsies from the caudal fin of adult fish were removed with a sharp blade after anesthesia with MS222. Embryos or fin clips were placed individually in lysis buffer, consisting of 1M KCl, 1M MgCl_2_, 1M Tris pH 8.3, NP40, Tween-20 and gelatine, supplemented with proteinase K to a concentration of 100 μg/ml. Lysis was performed for 1 h at 60 °C, 15 min at 95 °C, and then held at 4 °C. After adding 80 μl of ultrapure H_2_O (Milli-Q, Merck Millipore) lysates were used for PCR.

**Fig. 2:**
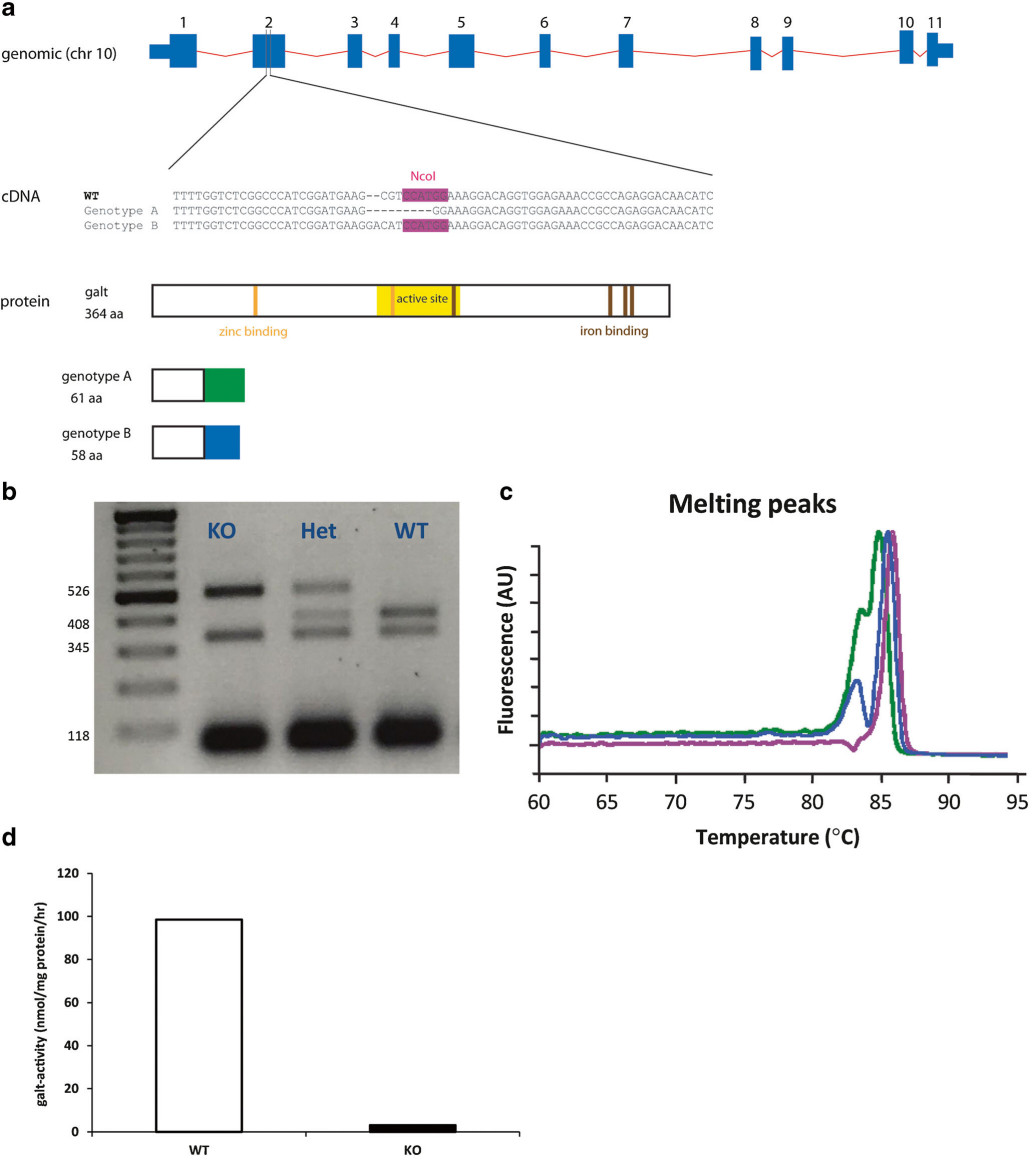
Establishment of a *galt* knockout model. Panel **a** shows the genomic layout of the zebrafish *galt* gene. Injection of galt-specific TALEN constructs, targeting exon 2, resulted in two different genotypes (A and B), both leading to a predicted frame-shift and a premature stop codon. Full length wildtype galt consists of 364 amino acids. Mutant protein in genotype A consists of the first 61 amino acids of the wildtype protein, followed by 26 amino acids that differ from wildtype galt. Mutant protein in genotype B consists of the first 58 amino acids of the wildtype protein, followed by 29 amino acids that differ from wildtype galt. The two genotypes of *galt* knockout zebrafish could be identified by NcoI, T7 endonuclease I and HRM analysis. Panel **b** illustrates a representative pattern of NcoI analysis of genotype A. Panel **c** illustrates a representative pattern for HRM analysis for genotype A or B (*pink*: wildtype, *blue*: heterozygous, *green*: knockout). Panel **d** shows a severe impairment of the galt catalytic activity in larval wildtype and knockout zebrafish (5dpf) using a HPLC assay

PCRs encompassing the targeted region of *galt* were conducted using Phusion Hot Start II DNA Polymerase (Thermo Scientific) in 50 μl reactions with 5 μl lysate per reaction as template. The following primers were used (including M13 sequence): forward primer 5′-TGTAAAACGACGGCCAGTATCGTTTGAAGCCAAAATCG-3′, reverse primer 5′- CAGGAAACAGCTATGACCTGCGTATTTCTCTGGATTTGC-3′. The PCR product was verified on a 1% agarose gel; 15 μl of the PCR product were combined with 0.5 μl NcoI restriction enzyme. Reaction mixes were incubated at 37 °C for three hours and then analyzed on a 3% agarose gel. Mismatches at the targeted region of *galt* were expected to result in loss of an NcoI restriction site, thereby generating two large fragments (526 and 345 bp) rather than three shorter fragments (408, 118, and 345 bp) (Fig. 2). The presence of a mutation in only one *galt* allele will therefore lead to both wild type and mutant fragments (526, 408, 345 and 118 bp).

Mutations positive by NcoI digestion were confirmed by Sanger sequencing.

Of PCR product 15 μl were denatured and re-annealed in a thermocycler by incubation for 2 min at 95 °C, for 10 s at 85 °C, for 15 min at 25 °C, and then held at 16 °C. The re-annealed products were treated with 0.5 μl of T7 endonuclease I (New England Biolabs) upon addition of 3 μl of buffer and 11.5 μl of H_2_O (Milli-Q, Merck Millipore) to a final volume of 30 μl. Reaction mixes were incubated for 20 min at 37 °C and analyzed on a 3% agarose gel. Mismatches at the targeted region of *galt* were expected to result in cleavage of heteroduplexes, thereby generating additional fragments. Mutations positive by T7 endonuclease I were confirmed by Sanger sequencing.

To enable large-scaled genotyping, a high resolution melting (HRM) analysis was applied (Wittwer et al 2003). Primers were designed using Primer3 software (Untergasser et al 2012) (forward primer 5′-TACAATCCTCTGCGGGACTC-3′; reverse primer 5′-CGTGGGATGTTGTCCTCTG-3′). Amplification and HRM analysis were performed in the LightCycler ®480 (Roche Applied Science), using the High Resolution Melting Master kit (Roche Applied Science). Both genotype A and genotype B amplicons were analyzed according to the following conditions: 95 °C for 10 min; 45 cycles of 95 °C for 10 s, 59 °C for 15 s, 72 °C for 15 s; one cycle of 95 °C for 60 s, 40 °C for 60 s, and a melting from 60 to 95 °C rising at 0.02 °C per second. All samples were run in triplicate. HRM analysis was performed using the software LightCycler ® 480 Gene Scanning version 1.5.1.

### Gene expression analysis RT-PCR

RNA was extracted from single adult organs using TRIzol (Life Technologies). A qScript cDNA Synthesis Kit (Quantabio) was used to generate cDNA. Quantitative real-time PCR (qPCR)-based quantification of *galt*, *galk1 (galactokinase)*, *gale (UDP-galactose 4′-epimerase)*, and *akr1b1 (aldose reductase)* expression was conducted, with *ef1a* as a reference gene (Tang et al 2007). Primer sequences are available on request. SYBR® green (Bioline) was used for all qPCR experiments. Samples, obtained from two independent RNA extractions were measured five times in total.

### Zebrafish galt activity

The galt activity was analyzed in embryos (5 days post fertilization, dpf) and adult fish. Thirty zebrafish embryos were suspended in 300 μL lysis buffer containing 80 mM Tris pH 8.0 and cOmplete mini EDTA-free Protease Inhibitor (Roche). The suspension was homogenized in a potter tube (10 strokes) and subsequently sonicated by the ultrasonic processor UP50H (Hielscher) with a 2 mm diameter tip, amplitude 175 μm, power density 480 W/cm^2^. Lysates were centrifuged at 11,500 × *g* for 20 min at 4 °C. 50 μL of the supernatant, diluted to contain 30 μg of protein (assessed with BCA Protein Assay Kit), were mixed with 0.4 mM dithiothreitol, 30 mM Gal-1-P, 125 mM glycine and 15 mM UDP-glucose (UDP-Glc), and subsequently incubated for 60 min at 28 °C. The reaction mix was further processed and analyzed by HPLC as described previously (Lindhout et al 2010). Moreover, two reaction mixes were used as controls, one containing 0.4 mM dithiothreitol, 125 mM glycine, and 15 mM UDP-Glc (no Gal-1-P), and the other containing 0.4 mM dithiothreitol, 30 mM Gal-1-P, and 125 mM glycine (no UDP-Glc). Gal-1-P, UDP-Glc, UDP-Gal, dithiothreitol, and glycine were purchased from Sigma-Aldrich/Fluka (Zwijndrecht, the Netherlands).

Additionally, galt activity was analyzed in adult fish (brain, ovary, and skeletal muscle) through a liquid chromatography tandem mass spectrometry (LC-MS/MS) as previously described (Li et al 2011). Briefly, incubation medium consisted of 16 μL of 0.5 M glycine buffer pH 8.7, 8 μL of 2 mM UDP-Glc, 8 μL of 2 mM ^13^C_6_-Gal-1-P, and 8 μL of 4 mg/mL tissue lysate and incubated at 30 °C water bath for 30 min. All samples were prepared in duplicate and measured twice. ^13^C_2_-Gal-1-P and ^13^C_6_-Gal-1-P were purchased from Omicron Biochemicals Inc. (South Bend, IN, USA). Glycine (culture grade) was purchased from Fisher Scientific (Waltham, MA, USA). All other chemicals were obtained from Sigma (St. Louis, MO, USA).

### Zebrafish galk1 activity

Catalytic galk1 activity was measured in ovarian samples. Gonads were added to a tube containing PBS buffer, 1% proteinase inhibitor cocktail (Sigma-Aldrich), and lysed by sonication at 4 °C. Protein measurement, galk1 assay, and LC-MS/MS were performed as previously described (Li et al 2014), except that 1 mg/mL ovarian lysate were added to the assay co-factors cocktail and incubated at 30 °C for 30 min. Briefly, galk1 assay consisted of 12.5 μL of 2 mM ^13^C_6_-galactose, 12.5 μl of 0.8 mol/L MOPS buffer pH 7.8, 12.5 μL mixture of co-factors cocktail (30 mM ATP, 32 mM MgCl_2_ and 32 mM NaF) with 25 μl of 6 mg/mL ovarian lysate. Two standard tubes were then put in 30 °C water bath for 1 h and 100 °C water bath for 3 min. One blank tube was put in 100 °C water bath for 3 min and 30 °C water bath for 1 h. After finishing incubation 20 μL of 100 μM internal standard, ^13^C_2_ Gal-1-P, with 96 μL acetonitrile were added to the tube. It was roughly vortex for 30 s and centrifuged at 14,000 × *g* for 10 min, 50 μL of supernatant was added to autosampler vial which contained 950 μL 80% acetonitrile for LC-MS/MS analysis. Calibrator concentrations of galk1 were 200, 100, 33.3, 11.1, 3.7, and 1.23 μmol/L.

Liquid chromatography, mass spectrometry (MS), column, and data analysis were similar to gale method but with different transitions and LC-MS settings. All samples were prepared in duplicate and measured twice. ^13^C_6_-Gal was purchased from Omicron Biochemicals Inc. (South Bend, IN, USA).

### Zebrafish gale activity

Frozen tissues were added to a tube containing 0.5 mol/L glycine pH 8.5 (Sigma-Aldrich), 1% proteinase inhibitor cocktail (Sigma-Aldrich), and lysed by sonication at 4 °C. Protein measurement, gale assay, and LC-MS/MS settings were the same as previously described (Li et al 2014) except that 20 μl of 4 mg/ml of brain or 1 mg/ml ovarian lysate were added to the cocktail and incubated at 30 °C for 30 min. ^13^C_3_-UDP-Glc (Sigma-Aldrich) was added as internal standard. All samples were prepared in duplicate and measured twice.

### Zebrafish ugp activity

The ugp activity was measured in brain, ovary, and skeletal muscle, in a reaction mixture consisting of 4.35 μL of 0.5 M glycine buffer pH 8.7 (Sigma-Aldrich), 4.35 μL of 0.66 mM UTP, 4.35 μL of 4 mM ^13^C_6_ Glc-1-P, 1.96 μL of 6 mM MgCl_2_, and 5 μL of 1 mg/ml tissue lysate and incubated at 30 °C for 3 min. The reaction was stopped by adding 45 μL of a mixture composed of 7.8 μL of 20 μM ^13^C_3_ UDP-Glc, 31.2 μL of acetonitrile, and 6 μL of 0.6 M formic acid. After mixing and centrifugation at 14000 × *g* for 10 min, 30 μL of the supernatant was used for LC-MS/MS analysis. Calibrator concentrations of ugp were 111, 37, 12.3, 4.1, 2.05, and 1.03 μmol/L. All samples were prepared in duplicate and measured twice. All chemicals and reagents were purchased from Sigma-Aldrich.

### Gal-1-P evaluation

In order to measure Gal-1-P in zebrafish, embryos were suspended in 300 μl lysis buffer containing 80 mM Tris pH 8.0 (Sigma-Aldrich) and Complete mini EDTA-free Protease Inhibitor (Roche). The suspension was homogenized in a potter tube (10 strokes) and subsequently sonicated by the ultrasonic processor UP50H (Hielscher) with a 2 mm diameter tip, amplitude of 175 μm, and power density of 480 W/cm^2^. Lysates were centrifuged at 11,500 × *g* for 20 min at 4 °C. Gal-1-P content was then determined in 70 μl of the supernatant, by a coupled-enzyme assay, as described in (Bosshard and Steinmann 2008). Briefly, in the first reaction, Gal-1-P is converted into galactose by alkaline phosphatase (AP), and in the second reaction, galactose is converted into galactone by galactose dehydrogenase (GDH). In the second reaction, NAD^+^ is used as a co-substrate by GDH, thus leading to the production of NADH, which was determined UV-spectrophotometrically (λ = 340 nm).

Accordingly, Gal-1-P quantification was carried out using two mixes: the first containing 160 mM Tris pH 8.6, NAD^+^ 12.5 mM, 43,000 U/ml AP and 60 U/ml GDH (galactose and Gal-1-P), and the second containing 160 mM Tris pH 8.6, NAD^+^ 12.5 mM and 60 U/ml GDH (galactose). A mix containing no enzyme was also prepared and used as blank. Gal-1-P concentration was calculated by subtracting total galactose (determined from reaction 2) to total galactose and Gal-1-P (determined from reaction 1), and normalized to zebrafish and protein content. Total protein was quantified using the BCA Protein Assay Kit (Thermo Scientific) (Smith et al 1985).

### Development and survival evaluation

Development and survival of all fish was compared between genotypes from 5 dpf until adulthood, in the absence of exposure to galactose. At the age of 5 dpf morphology was studied with microscopy. Specific attention was paid to hatching and swim bladder development. Abnormal fish were genotyped to evaluate whether the three different genotypes (wildtype, heterozygous, knockout) were disproportionally present in these fish. Assessment of survival rates, and comparison between genotypes, was conducted on a daily basis throughout the embryonic phase, and monthly thereafter.

### Galactose challenge

Wildtype and knockout embryos (*n* = 30 per genotype) of 24 h post fertilization (hpf) were exposed to different concentrations of galactose-containing E3 medium (50–300 mM) in 100 mm petri dishes. Unexposed wildtype and knockout embryos (*n* = 30 per genotype) grown in E3 medium were used as a control. All animals were monitored daily for viability until they reached 5 or 9 days of age.

### Fertility evaluation

A selected pool of unchallenged, adult age-matched *galt* wildtype (*n* = 24; 12 fish per tank), heterozygous (*n* = 12; two tanks of 6 fish plus 6 TL fish, total of 12 fish per tank) and knockout zebrafish (*n* = 24; 12 fish per tank) (*n* = 60) was crossed periodically (total of 10 crossings), to explore fertility in the different genotypes. The following crossings were conducted (six pairs per condition): wildtype incross, heterozygous incross, knockout incross, wildtype female crossed to knockout male, and knockout female crossed to wildtype male. This sample size was determined as the maximum number of crossings which was practically possible. Mass mating of wildtype and knockout fish (five pairs per genotype; total of five crossings) was also performed. The number of eggs and egg quality per spawning event per genotype were assessed. Egg quality was assessed immediately after spawning and at 24 hpf to evaluate initial egg quality and fertilization rate, respectively. Inclusion criteria for fertility analysis; successful crossing was defined as an event where >10 fertilized eggs were produced per pairwise mating. This criterion was established before experiments were started.

### Neurological assessment

Zebrafish embryos (5 dpf) and juveniles (4 weeks old) were placed in 24- and 12-well plates, respectively. Motor activity was quantified using a ZebraBox (Viewpoint) device under exposure to light for a period of 60 min. Activity is expressed as an average number of changes in pixels (arbitrary units) throughout the experiment. Motor activity was evaluated and compared between genotypes.

### Statistical analyses

Levene’s test was used as a preliminary screen for homogeneity of variances between or among groups with equal *n* values and the Brown–Forsythe test was used for groups with unequal *n* values. Differences between two groups were evaluated for statistical significance using an independent t-test (two-tailed), and differences between more than two groups were evaluated for statistical significance by one-way analysis of variance (ANOVA).

The number of unsuccessful crossings exhibited by each knockout group was compared with that of wildtype pairs by chi square analysis (Fisher’s exact test, double-sided). Egg quality was evaluated by quantifying the amount of viable and unviable eggs immediately after spawning and at 24 hpf (unsuccessful crossings excluded), and the ratio of the average viable eggs per mating was compared between groups by non-parametric, one sample analysis.

For all tests a *p* value less than 0.05 was considered statistically significant.

## Results

### Zebrafish metabolize galactose via the Leloir pathway

To explore the role of galactose metabolism in zebrafish, we first confirmed that the zebrafish genome encodes and expresses orthologues of the human genes encoding the Leloir pathway enzymes (GALK1, GALT, GALE) and the enzymes involved in escape routes (AR, UGP) (Fig. 1). A BLAST search identified a single zebrafish orthologue of human *GALT* on chromosome 10 of the zebrafish genome (ENSDARG00000069543). The zebrafish *galt* gene has two protein coding transcripts, of which the first (galt-001, ENSDART00000138161) encodes a protein of 364 amino acids and shows the strongest conservation with the human orthologue (73%) (Fig. 1). Despite the fact that fish underwent a genome duplication throughout evolution (Howe et al 2013), the *UGP2* gene is the only duplicated gene related to galactose metabolism in zebrafish (Fig. 1).

### *galt* TALEN-generated mutants are loss-of-function

TALEN mRNA pairs targeting exon 2 of *galt* were injected in AB wildtype zebrafish embryos in the one-cell stage. A concentration of 150 pg of both TALEN mRNAs were injected after assessing toxicity and as a function of injection dose at 24 hpf. NcoI and T7 endonuclease I genotyping, followed by Sanger sequencing confirmation, revealed the generation of two *galt* mutant genotypes (Fig. 2a and b). Genotype A (c.106_112delCGTCCAT) represents a 7 bp deletion, leading to a frame-shift at position c.106 and a subsequent introduction of a premature stop codon 26 amino acids downstream; the mutation is positive by NcoI and T7 endonuclease I analyses (Fig. 2b; Supplementary Fig. S2). Genotype B (c.[106_107delCG; 106_109insGACA]) consists of a 2 bp deletion and 4 bp insertion, leading to a frame-shift at position c.106 and introduction of a premature stop codon 29 amino acids downstream; the mutation is negative by NcoI digestion and positive by T7 endonuclease I analysis. Genotype A and B presented the same melting profile by high resolution melting (HRM) analysis (Fig. 2c).

**Fig. 3:**
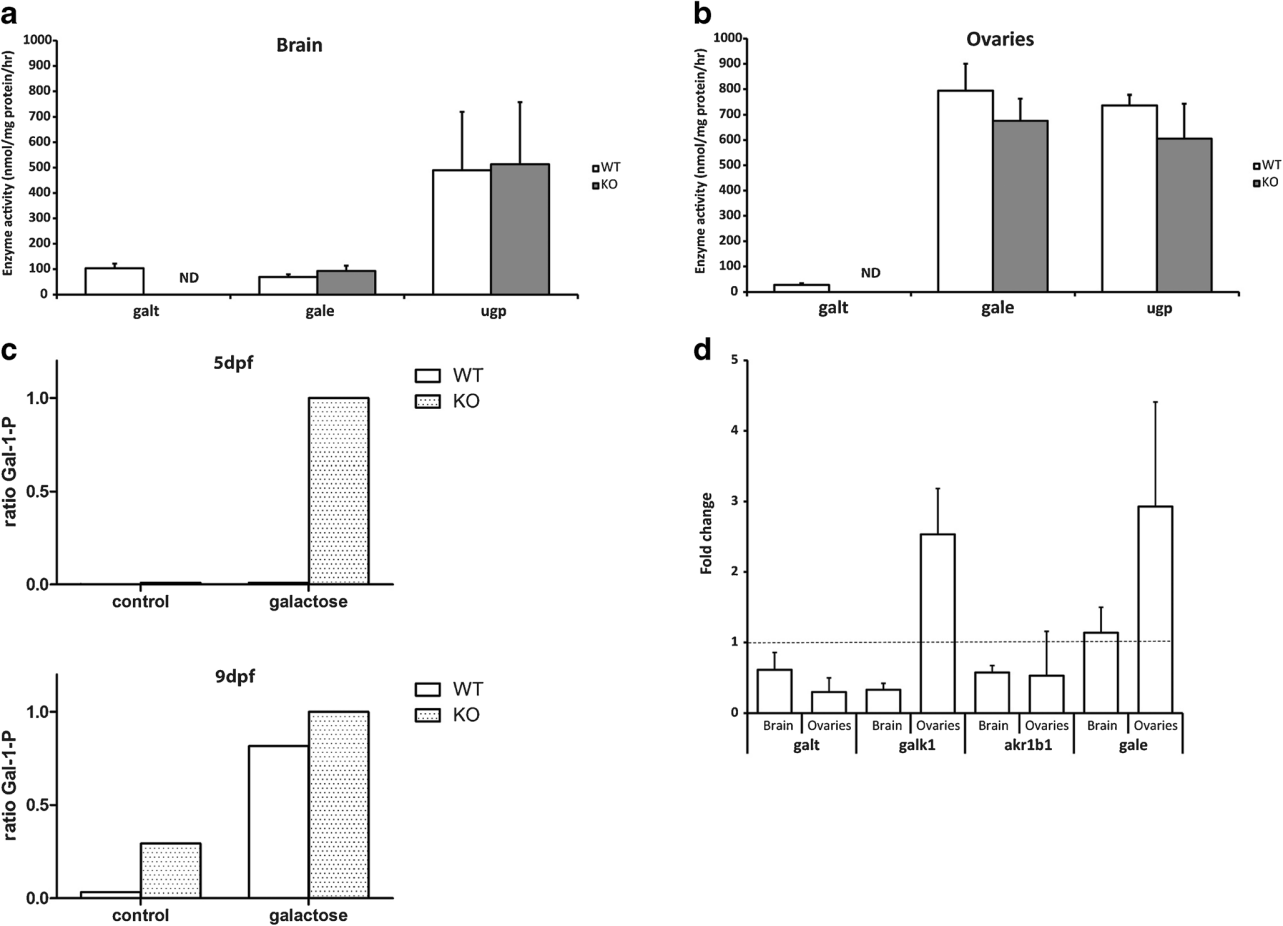
Biochemical phenotype of *galt* knockout zebrafish. Panel **a** and **b**: *galt* knockout fish demonstrated essentially null galt activity in adult brain (panel **a**) and ovaires (panel **b**) using an LC-MS/MS method. Enzyme activity of indicated enzymes is plotted per genotype as the average activity +/− SD. ND: not detectable. Sample sizes were: in brain (panel **a**); galt activity wildtype and knockout: *n* = 5; gale activity wildtype *n* = 10 and knockout *n* = 8; ugp activity wildtype and knockout *n* = 5. In ovary (panel **b**); galt activity wildtype *n* = 5 and knockout: *n* = 4; gale activity wildtype and knockout *n* = 3; ugp activity wildtype and knockout *n* = 3. Panel **c**: When challenged with 200 mM of galactose for 5 days (1dpf-5dpf), knockout zebrafish accumulated high concentrations of Gal-1-P, whereas levels remained essentially null in wildtype zebrafish. Values are from two independent experiments (*n* = 2) and are presented as ratio of knockout Gal-1-P when exposed to galactose. Panel **d**: When challenged with 200 mM of galactose for 8 days (1dpf-9dpf), knockout zebrafish accumulated high concentrations of Gal-1-P, whereas levels remained low in wildtype zebrafish. Values are from two independent experiments (*n* = 2) and are presented as ratio of knockout Gal-1-P when exposed to galactose. Panel **e**: mRNA expression levels of 4 key enzymes of the Leloir pathway was evaluated by QPCR. Values are given as expression fold change of the knockout compared to the wildtype tissues. Measurements were performed 5 times, each in triplicate

The galt enzyme activity was essentially null in knockout embryos at 5 dpf (3.1%; Fig. 2d), confirming at the biochemical level that the knockout represents a loss-of-function allele. The galt enzyme activity was undetectable in the brain or ovary of adult knockout fish, the target organs of long term damage in classic galactosemia (Fig. 3a and b), as well as in skeletal muscle (Supplementary Fig. S3).

### *galt* knockout embryos accumulate Gal-1-P after exposure to galactose

To identify susceptibility to galactose, knockout embryos were challenged with different concentrations of galactose, of which a concentration of 200 mM galactose was chosen to proceed with the galactose challenge studies. In order to avoid inducing early developmental effects, embryos were exposed to galactose for 24 hpf onward till the ages indicated.

When exposed to exogenous galactose, knockout embryos accumulated high amounts of Gal-1-P at 5 dpf as compared to the unexposed situation and to wildtype fish (Fig. 3c).

At 9 dpf, knockout fish accumulated asignificant amount of Gal-1-P in the absence and presence of galactose. Wildtype fish presented low Gal-1-P levels in the absence of exogenous galactose, whereas in the exposed situation accumulated higher Gal-1-P levels (Fig. 3d).

### galk1, gale, ugp activity

Catalytic activity of galk1, gale, and ugp—other enzymes of galactose metabolism—was also measured in adult fish. The galk1 activity was measured in the ovary, which revealed no statistically significant difference between knockout and wildtype fish (*p* = 0.83). The gale activity measured in the brain and ovary, and was shown to be marginally elevated in brain (*p* = 0.16) and slightly reduced in the ovary of knockout animals (*p* = 0.21) comparatively to wildtype. The gale enzyme activity was 8–10 fold higher in the ovary than in brain, both in knockout (*p* = 0.0056) and wildtype animals (*p* = 0.0069). Activity of ugp was also not significantly different in knockout versus wildtype ovary and brain (*p* = 0.26 and *p* = 0.38, respectively), with no clear difference in activity between these two tissues (Fig. 3a and b). Muscle ugp activity revealed that the knockout fish had more than 2 times higher that of the wildtype animals (*p* = 0.034; Supplementary Fig. S3). In addition, we assessed mRNA expression levels in adult brain and ovary of knockout animals vs. controls (Fig. 3e). Transcriptional analysis revealed *galk1* and *gale* mRNA were more than two-fold higher in ovary of knockout fish (*p* = 0.0014 and *p* = 0.025, respectively).

### Normal development and growth

Unchallenged *galt* knockout fish undergo normal development and growth: hatching, swim bladder development, morphology or survival rate are not affected throughout embryo, juvenile or adult stages. Thus, *galt* knockout zebrafish are morphologically indistinguishable from heterozygotes and wildtype fish; growth and survival rates are similar (data not shown).

### Fertility evaluation

Heterozygous pairs did not exhibit a statistically significant difference in egg quantity (*p* = 0.106; 95% confidence interval was of −73.0 ± 44.6) or quality as compared to wildtype pairs (*p* = 0.228; 95% confidence interval was of −38.9 ± 31.9).

**Fig. 4:**
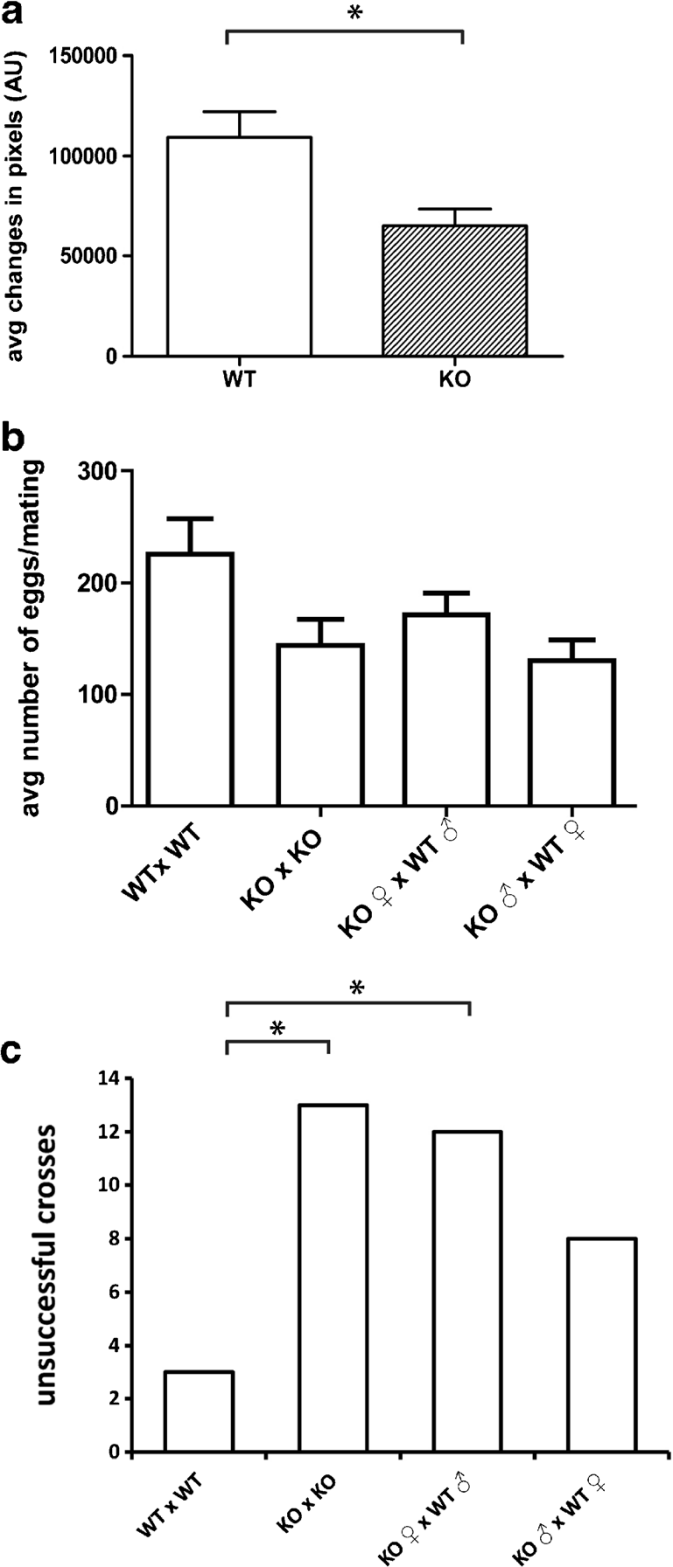
Chronic impairments. Impairments of motor activity and fertility were observed in *galt* knockout zebrafish never exposed to exogenous galactose. Reduced motor activity was observed in knockout juvenile fish (4 weeks old) as compared to matched controls (*n* = 66 per genotype; motor activity expressed as mean ± SEM from two independent measurements) (panel **a**). Adult knockout pairs, wildtype female/knockout male and knockout female/wildtype male exhibited a lower average egg quantity per mating as compared to wildtype pairs (panel **b**; expressed as mean ± SEM from 10 independent crossing events). The number of unsuccessful crossings was higher in the presence of a knockout female (panel **c**)

Knockout pairs, wildtype female/knockout male, and knockout female/wildtype male exhibited a lower egg quantity per mating as compared to wildtype pairs (Fig. 4b; *p* = 0.044).

The number of unsuccessful crossings revealed a statistically significant difference between knockout pairs and knockout female/wildtype male comparatively to wildtype pairs (*p* = 0.012 and *p* = 0.022, respectively). The number of unsuccessful crossings was not statistically significantly different between wildtype female/knockout male and wildtype pairs (*p* = 0.199). There was a higher frequency of unsuccessful crossings whenever a knockout female was involved (Fig. 4c).

Egg quality was analyzed by the average ratio of viable eggs at 24hpf, excluding unsuccessful crossings. Non-parametric, one sample analysis revealed that there is no statistically significant difference between the egg quality laid by any of the groups (*p* = 0.662). Egg quality seems to be unaffected in *galt* knockout fish (Supplementary Fig. S4).

Mass mating revealed a higher number of eggs in wildtype fish (Supplementary Fig. S5).

### Neurological assessment

Knockout embryos (5 dpf) showed similar activity as wildtype controls, both without (*p* = 0.472) and with (*p* = 0.114) prior exposure to exogenous galactose (data not shown). At a later stage, unchallenged 4 weeks old knockout juvenile fish showed a statistically significant lower activity than age-matched wildtype control (*p* = 0.004) (Fig. 4a).

## Discussion

Herein we describe the successful development and characterization of a zebrafish model for classic galactosemia.

The *galt* knockout zebrafish mimics the human phenotype at both the biochemical and clinical levels. Enzymatic assays confirmed successful loss-of-function, with essentially null galt activity. Additionally, knockout fish accumulated elevated concentrations of Gal-1-P, which was exacerbated upon exposure to exogenous galactose. Catalytic activity of galk1, gale, and ugp were also measured, and interestingly varied considerably in the various tissues, which probably reflect specific needs of the given organ (Adeva-Andany et al 2016; Coelho et al 2017; Walter and Fridovich-Keil 2014). In contrast with their specific activity which was essentially the same as in wildtype fish, *galk1* and *gale* mRNA were significantly higher in ovary of knockout fish, suggesting that specific cellular signaling in knockout fish possibly increased *galk1* and *gale* transcription, repressed galk1 and gale synthesis and/or increased galk1 and gale turnover.

Whereas knockout larvae exhibited a motor activity indistinguishable of that of wildtype larvae, on the long term, knockout fish showed a significantly decreased motor activity as compared to that of wildtype fish, despite no prior exposure to exogenous galactose. Furthermore, the reproductive capacity in adult knockout zebrafish without previous exposure to exogenous galactose was decreased as compared to wildtype fish, presenting a lower egg quantity and a higher frequency of unsuccessful crossings. Even though to a lower extent than female fish, knockout male fish also appear to have impaired fertility. The decrease in motor activity might to some extent have influenced mating behavior and indirectly also affected the reproductive capacity.

These findings are in line with the human phenotype, in which patients present a severely impaired GALT activity and accumulate high levels of Gal-1-P, particularly in the newborn period upon exposure to galactose-containing milk (Schadewaldt et al 2003). Regarding the chronic complications, the observed brain and gonadal impairments in unexposed galt knockout zebrafish are in line with the human phenotype of neurological and ovarian sequelae, which occur even in patients that have had galactose restriction from their first day of life (Hughes et al 2009; Rubio-Agusti et al 2013; Waisbren et al 2012). Impaired male fertility in fish is in line with previous reports of a possible not clinically relevant male fertility impairment (Gubbels et al 2013).

Moreover, the clinical findings in our zebrafish model are in line with observations in other animal models for classic galactosemia. GALT-deficient flies exhibited motor impairments (Kushner et al 2010), whereas GALT-deficient mice exhibited brain abnormalities, and females showed a smaller litter size and longer time to achieve pregnancy (Chen et al 2017; Tang et al 2014).

With the creation of our zebrafish line, we have generated a model that complements these existing models by allowing the study of organ development from embryo stage to adulthood, which can provide important new insights on when lesions of target organs occur in galactosemia. Hitherto, it remains to be elucidated whether organ toxicity has a prenatal or postnatal onset, or extends over multiple developmental stages. Though the fly and mouse models already demonstrated great importance for pathogenesis studies, the zebrafish model is more amenable to organ studies throughout development, which are essential to answer the long-standing open question if, and to what extent, prenatal organ damage occurs. The availability of transgenic reporter lines and accessibility to sophisticated imaging techniques allow studying folliculogenesis and neurogenesis from embryonic stage to adulthood. In addition, the high-throughput screening potential of our model facilitates rapid and efficient testing of pharmacologic compounds.

In conclusion, we have successfully developed a *galt* knockout zebrafish model, which phenocopies the human hallmarks of classic galactosemia at both the biochemical and clinical levels. Notably, we show that brain and gonadal impairments are present despite no previous exposure to exogenous galactose. We generated this model because of the unique features of the zebrafish that allow studies on onset of damage and development of new therapeutic strategies.

## Electronic supplementary material

Supplementary Fig. S1 (JPEG 3463 kb)

Supplementary Fig. S2 (JPEG 945 kb)

Supplementary Fig. S3 (JPEG 834 kb)

Supplementary Fig. S4 (JPEG 1701 kb)

Supplementary Fig. S5 (JPEG 5759 kb)
