# Supplementary figures and images for: Impaired fertility and motor function in a zebrafish model for classic galactosemia
Source: J Inherit Metab Dis. 2017 Sep 14;41(1):117–27. doi: 10.1007/s10545-017-0071-1 (PMC5786655; doi:10.1007/s10545-017-0071-1)

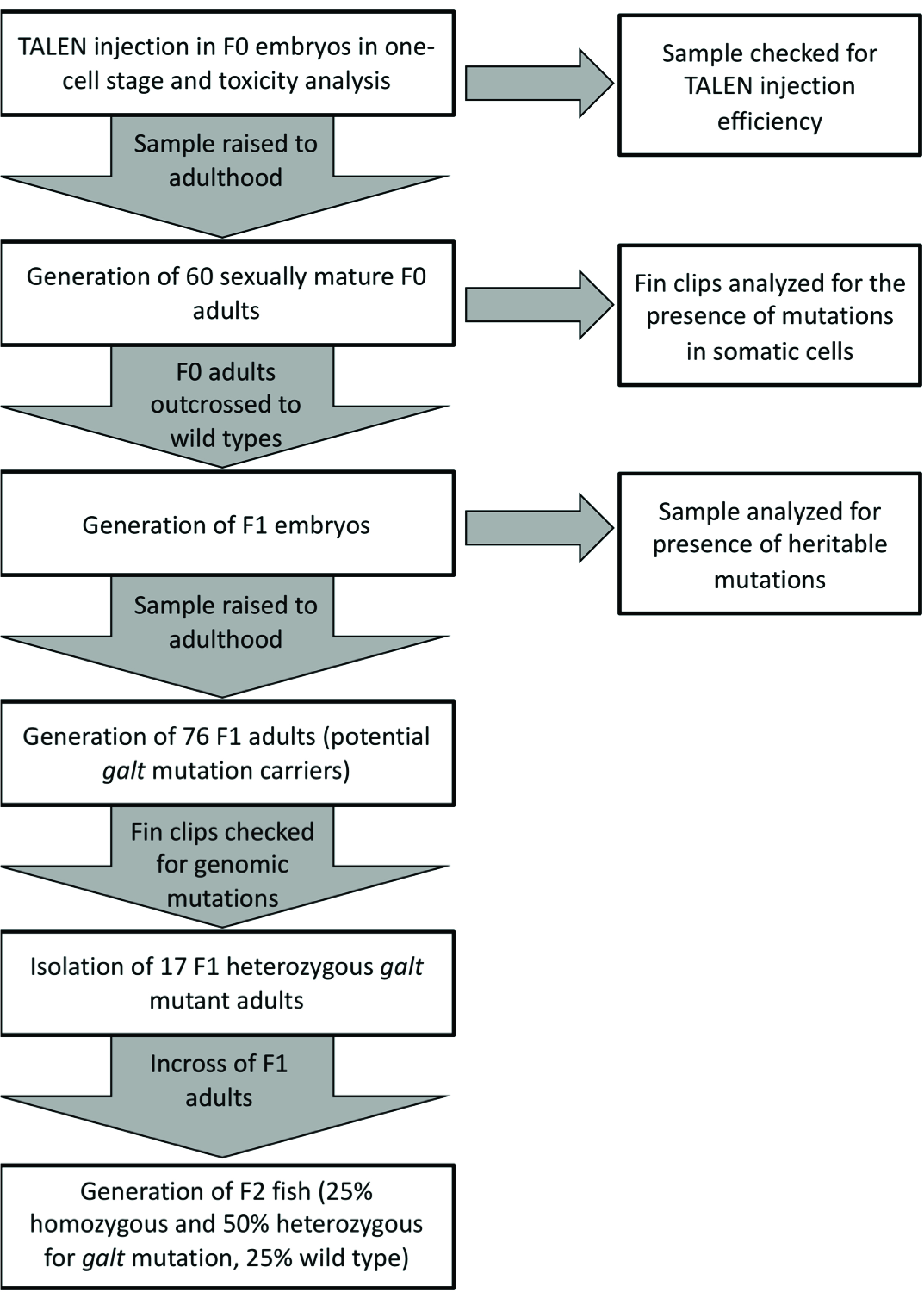

Supplement: Supplementary file 1 — (JPEG 3463 kb) [file 10545_2017_71_MOESM1_ESM.jpg]

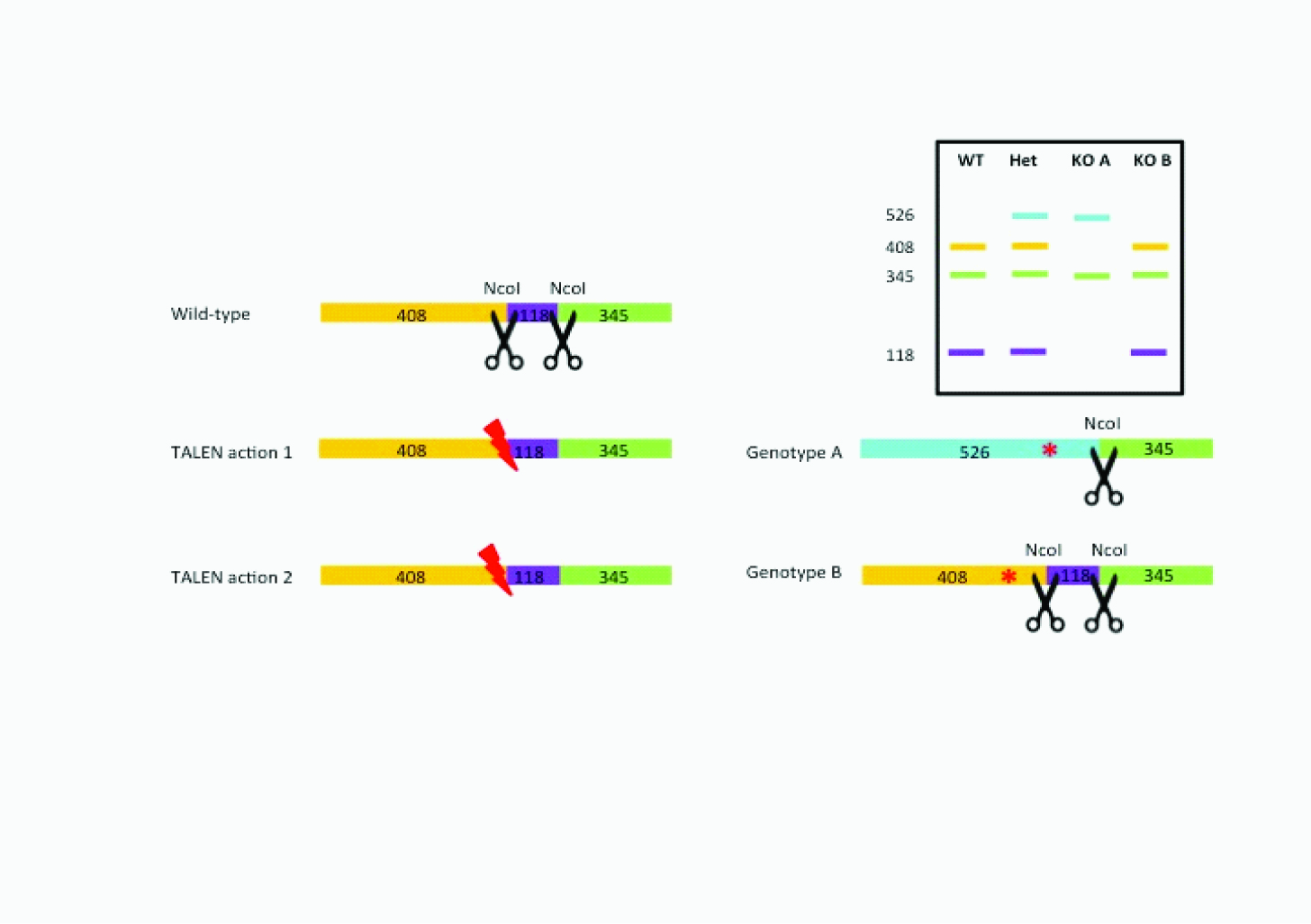

Supplement: Supplementary file 2 — (JPEG 945 kb) [file 10545_2017_71_MOESM2_ESM.jpg]

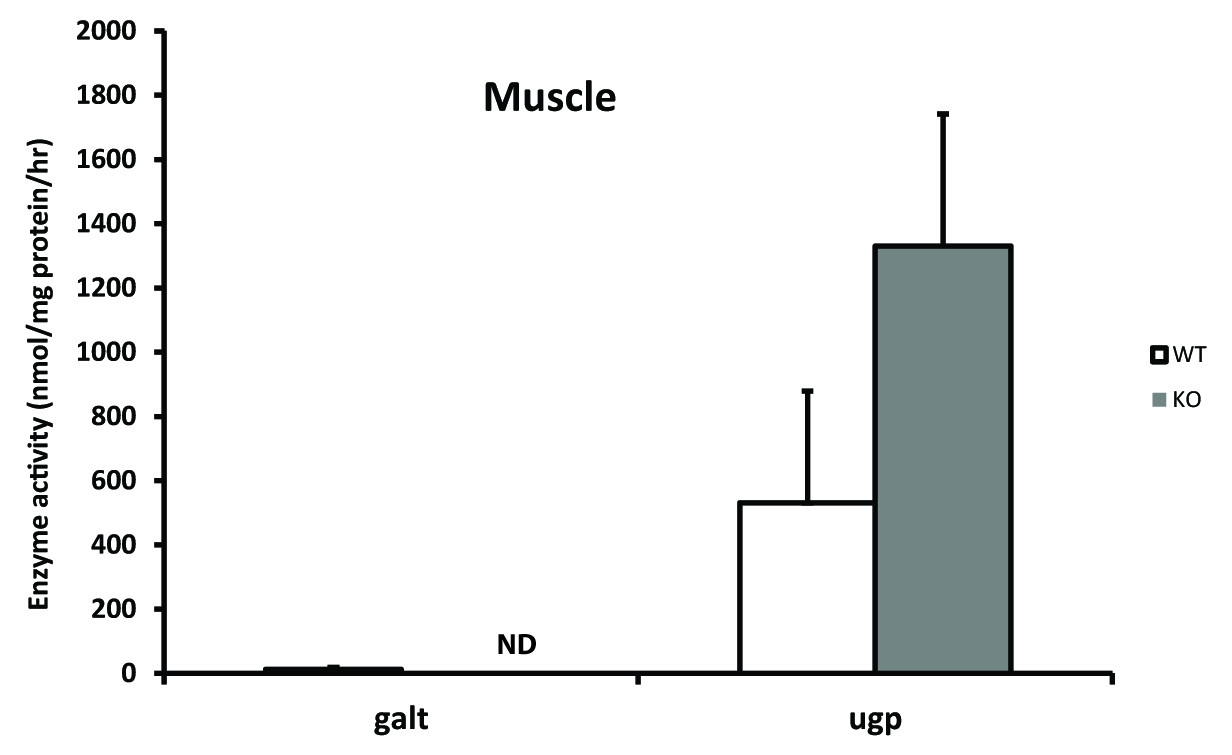

Supplement: Supplementary file 3 — (JPEG 834 kb) [file 10545_2017_71_MOESM3_ESM.jpg]

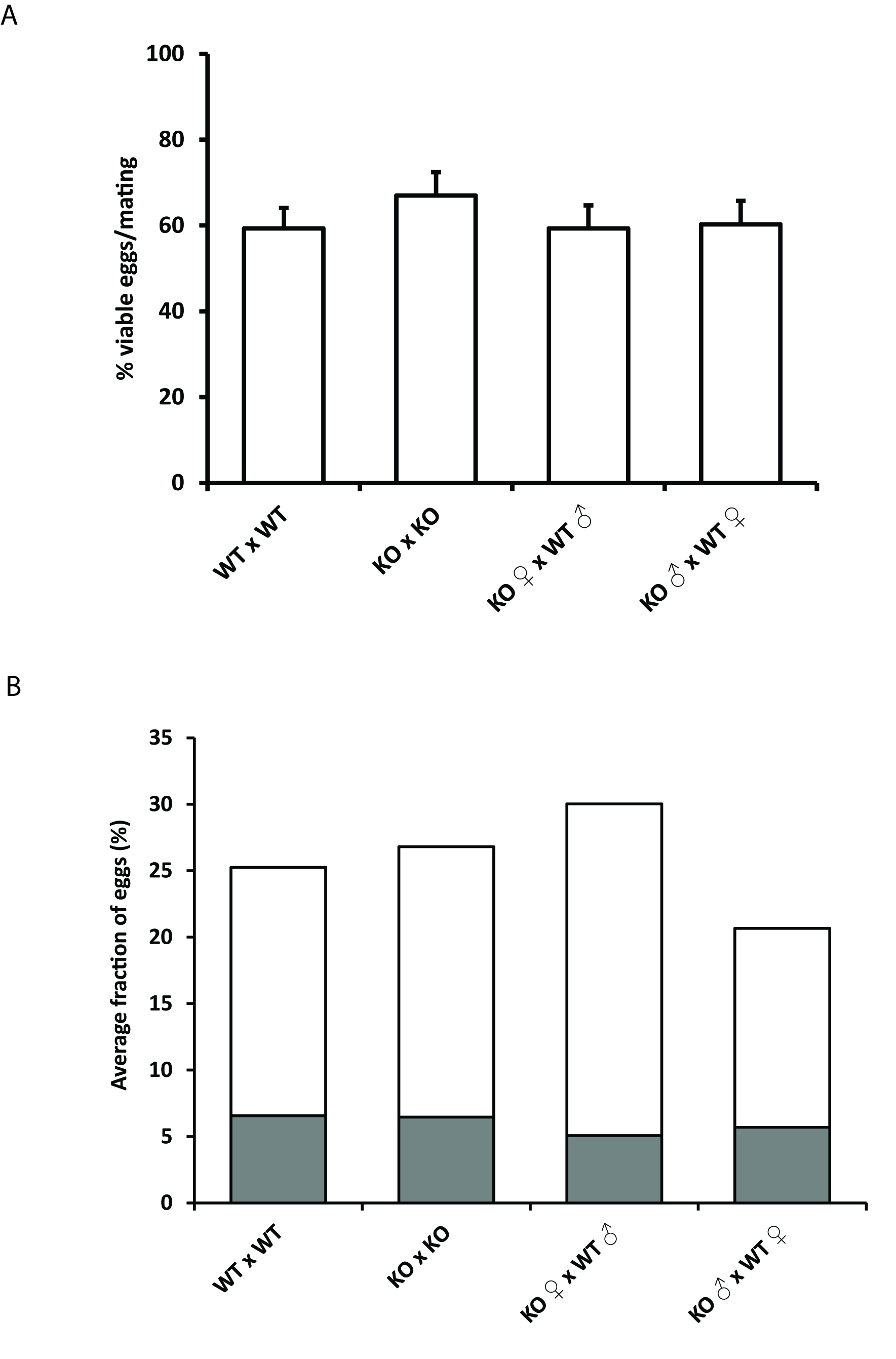

Supplement: Supplementary file 4 — (JPEG 1701 kb) [file 10545_2017_71_MOESM4_ESM.jpg]

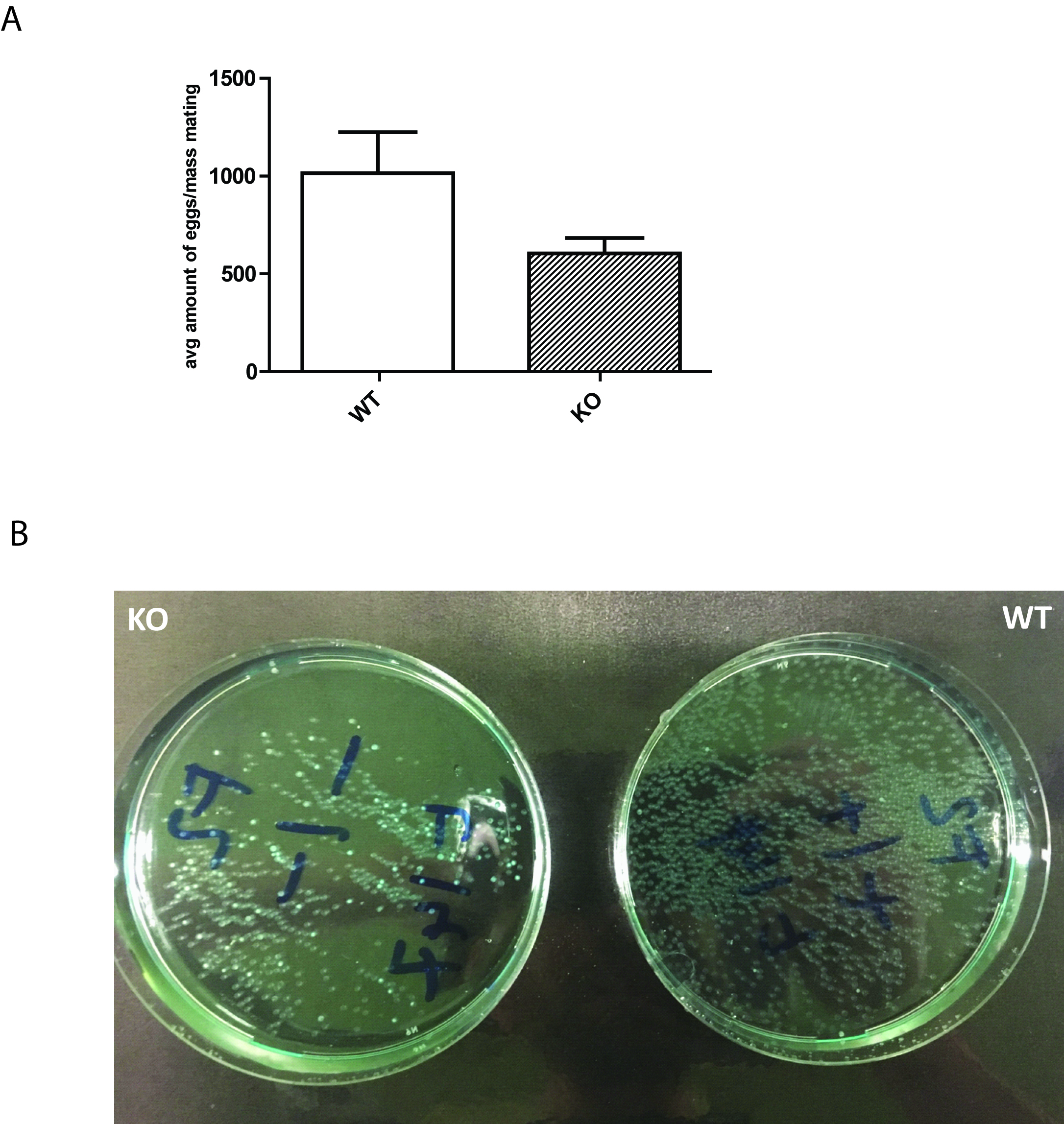

Supplement: Supplementary file 5 — (JPEG 5759 kb) [file 10545_2017_71_MOESM5_ESM.jpg]
